# Evidence supporting oxidative stress in a moderately affected area of the brain in Alzheimer’s disease

**DOI:** 10.1038/s41598-018-29770-3

**Published:** 2018-08-01

**Authors:** Priscilla Youssef, Belal Chami, Julia Lim, Terry Middleton, Greg T. Sutherland, Paul K. Witting

**Affiliations:** 1 Redox Biology Group, Discipline of Pathology, University of Sydney, Sydney, NSW 2006 Australia; 2 Neuropathology Group, Discipline of Pathology, The University of Sydney, Sydney, NSW 2006 Australia

## Abstract

The pathogenesis of Alzheimer’s disease (AD) remains to be elucidated. Oxidative damage and excessive beta-amyloid oligomers are components of disease progression but it is unclear how these factors are temporally related. At post mortem, the superior temporal gyrus (STG) of AD cases contains plaques, but displays few tangles and only moderate neuronal loss. The STG at post mortem may represent a brain region that is in the early stages of AD or alternately a region resistant to AD pathogenesis. We evaluated expression profiles and activity of endogenous anti-oxidants, oxidative damage and caspase activity in the STG of apolipoprotein ε4-matched human AD cases and controls. Total superoxide dismutase (SOD) activity was increased, whereas total glutathione peroxidase (GPX), catalase (CAT) and peroxiredoxin (Prx) activities, were decreased in the AD-STG, suggesting that hydrogen peroxide accumulates in this brain region. Transcripts of the transcription factor *NFE2L2* and inducible *HMOX1*, were also increased in the AD-STG, and this corresponded to increased Nuclear factor erythroid 2-related factor (NRF-2) and total heme-oxygenase (HO) activity. The protein oxidation marker 4-hydroxynonenal (4-HNE), remained unchanged in the AD-STG. Similarly, caspase activity was unaltered, suggesting that subtle redox imbalances in early to moderate stages of AD do not impact STG viability.

## Introduction

Alzheimer’s disease (AD) is the most common neurodegenerative disease. Its prevalence is increasing markedly worldwide due to ageing populations and a lack of disease modifying therapies. AD clinically manifests as memory deficits in combination with a progressive deterioration of other cognitive domains such as executive function and visuospatial skills.

At post mortem examination severely affected AD brains are commonly reduced to half their original volume^1^ with a disproportionate neuronal loss from the entorhinal cortex, amygdala, and hippocampus^2^. The pathology of AD is also characterized by two pathognomonic entities, intracellular neurofibrillary tangles (NFTs) and extracellular plaques. The major constituent of plaques is a collection of peptides called beta-amyloid (Aβ) while NFTs are largely composed of paired helical filaments of an abnormally hyperphosphorylated form of the microtubule associated protein tau (MAPT or tau). Tau pathology spreads in a predictable manner, from the allocortex to the association areas of the neocortex and lastly the primary cortices^3,4^. Cortical layers within the entorhinal cortex from severely affected subjects can exhibit up to 90% neuronal loss^5^ while regions such as the primary visual cortex remain essentially unaffected by the disease process with mild Aβ accumulation and minor alterations in neuronal form and functions.

The popular amyloid cascade hypothesis (ACH), suggests that the common sporadic forms of AD results from the accumulation of soluble oligomeric forms of Aβ in the neuropil. These toxic products disrupt neuronal kinase/phosphatase and redox balance, leading to tau hyperphosphorylation, NFT formation and neurodegeneration^6^. However, the mechanisms leading to the accumulation of Aβ oligomers in sporadic AD are not clear. Epidemiological studies have not been particularly informative with only ageing, the possession of the apolipoprotein E (*APOE*) ε4 allele, female gender and diabetes being consistently identified as risk factors^7^.

In addition to plaques and NFTs, the AD brain is also characterized by oxidative damage to proteins, nucleic acids^8^ and lipids^9^. It is generally accepted that Aβ may act directly as a pro-oxidant^10^ or indirectly by precipitating *NMDA* receptor-dependent Ca^2+^ influxes that result in mitochondrial dysfunction and the subsequent generation of reactive oxygen species (ROS)^11^. These, in turn, stimulate antioxidant response pathways that involve redox sensitive transcription factors *e.g*., NRF-2 (nuclear factor, erythroid 2-like 2; encoded by *NFE2L2*). Activation of NRF-2 is known to stimulate the potent inducible antioxidant hemeoxygenase-1 (HO-1) and possibly other endogenous antioxidant response elements^11^. Yet other researchers hypothesise that Aβ accumulation may be a consequence of oxidative stress^12^ and suggest that Aβ^13^ and indeed tau^14^ act as anti-oxidants in AD (reviewed by Sutherland *et al*.)^15^ thereby, potentially limiting the observed radical-mediated damage to DNA, proteins and lipids that occurs during the pathogenesis of this neurodegenerative disease^16,17^.

Notwithstanding that the outcomes from clinical trials of Aβ-modifying therapies in asymptomatic individuals are outstanding, there is still a need to pursue adjunctive or alternative therapeutic targets in AD, through accurate modelling of the early phases of the disease^18^. In this study, we explore markers of oxidative damage in the superior temporal gyrus (STG). The AD-STG at post-mortem shows moderate levels of insoluble tau and plaques while retaining the majority of its neurons^19^. Therefore, the post-mortem STG may represent a surrogate for the state of the most severely affected regions such as the entorhinal cortex, earlier in the disease course. An understanding of events in the post-mortem STG may allow a better temporal appreciation of linkages between oxidative stress and AD pathogenesis and more generally reveal novel pathways to exploit in the development of therapeutics to combat AD at a stage prior to irreversible cognitive decline.

## Results

### Detection of advanced Aβ but early tau pathology in AD-STG

**Figure 1:**
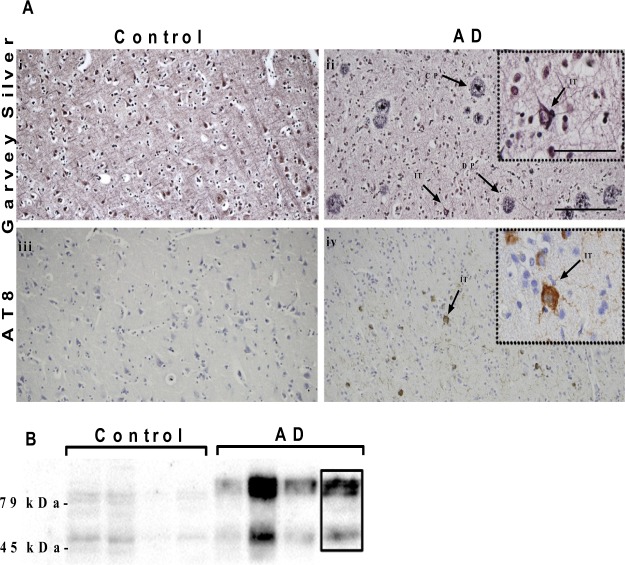
Pathology in the AD-STG. (**A**) (i–ii) Photomicrographs of Garvey silver staining from the STG of (i) a control showing no pathology and (ii) an AD case with numerous plaques and a single intracellular NFT (indicated by →). Scale bar in main images represents 20 μm. Scale bar to the insets represents 40 μm. (iii–iv) Photomicrographs of AT8 immunostaining from the STG of (iii) a control and (iv) an AD case with numerous tau-positive neurons including an intracellular NFT (indicated by →). CP = cored plaque; DP = diffuse plaque; IT = intracellular neurofibrillary tangle. (**B**) AT8 (tau) immunoblotting of whole brain homogenates showing hyperphosphorylated tau (positive bands in the range 45–79 kDa) in the AD samples (for complete image refer to Supplementary Fig. S1).

As previously described, the STG region of the AD cases (AD-STG) in this cohort showed typical AD pathology with significantly more plaques (both diffuse and cored) and AT8-positive neurons (Fig. 1A,B and Supplementary Table 1)^19^. Similarly, immunoblotting showed markedly increased AT8 tau-positive bands (between 45–79 kDa) in the AD-STG (Fig. 1B, refer to Supplementary Fig. S1). NFTs were relatively sparse, and neuronal counts were lower (16%) but not significantly different to controls (Supplementary Table 1). Taken together, the paucity of NFTs and minimal loss of neurons suggest that the disease, tau pathology in particular, is at an early stage in the AD-STG compared with the medial temporal lobe structures (Braak stage V/VI) from these same patients. AD cases also showed a significantly lower mean RNA integrity number (RIN) and brain pH and these two parameters were correlated with one another (r^2^ = 0.2, p = 0.002) (Supplementary Table 1). Although no differences in the mean fixation (storage) time (p = 0.06) between AD cases and controls were noted, fixation time was noted to range from 6 to 155 months. Additionally, a difference in post-mortem interval (PMI) was observed between the two groups (Supplementary Table 1; *p* = 0.04).

### Assessing anti-oxidant gene expression in AD and control STG

The disparity in mean RNA integrity number (RIN) identified between the cases and controls had the potential to skew gene expression analyses. In particular housekeeping genes need to reflect RIN differences between cases and controls rather than no difference as per normal^19^. The housekeeping gene Succinate Dehydrogenase Complex Flavoprotein Subunit A (SDHA) satisfied this modified criterion while displaying no difference between cases and controls in a RIN-matched subcohort (n = 21, data not shown). With SDHA as house-keeping gene, significant increases in both *NFE2L2* (encoding the transcription factor NRF-2) (*p* = 0.008) and *HMOX1* (encoding HO-1, the inducible form of HO) (p = 0.009) mRNA were determined in the AD-STG (Table 1). In contrast, there were no differences in *HMOX2* (encoding the constitutively expressed form of HO, HO-2) or *SOD1* mRNA with the latter varying widely in the AD-STG.

**Table 1:** Summary of STG gene expression in AD and control samples^a^.

Gene	Control	AD	p-value
*HMOX1*	0.09 ± 0.07, n = 17	0.30 ± 0.30, n = 20	0.009
*HMOX2*	6.50 ± 3.50, n = 17	11.10 ± 11.20, n = 20	0.15
*NFE2L2*	0.40 ± 0.30, n = 18	1.30 ± 1.20, n = 19	0.008
*SOD1*	36.04 ± 24.9, n = 18	36.04 ± 24.90, n = 19	0.16

^a^Levels of mRNA in STG brain homogenates were assessed by RT-qPCR as described in the methods section Note, for HMOX 1 and HMOX 2, two controls and one case was removed due to non-compliant melt curve. For *NFE2L2* and SOD 1, two samples failed to generate PCR products (due to low quality RNA) and one other sample was removed due to a failure in obtaining a PCR product for SHDA (hence normalizing to the house keeping gene was not possible in this case).

### Anti-oxidant protein levels and activity

**Figure 2:**
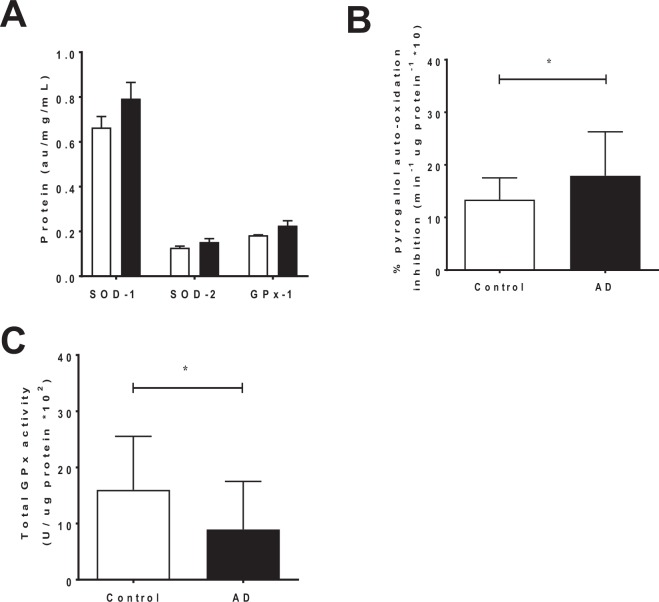
SOD and GPX protein expression and activity in the STG of AD cases and controls. Sample supernatants displaying (**A**) Quantification of SOD1, SOD2 and GPX1 protein expression by direct (**B**) Total SOD activity, reflected by the inhibition of pyrogallol auto-oxidation as a % of a control lacking the presence of SOD, and (**C**) Total GPX activity, assessed by monitoring the consumption of NADPH. Data represent mean ± SD, n = 19 for control and n = 20 individual AD cases; *p < 0.05.

There was no difference in SOD1, SOD2 and GPX1 protein levels between AD and control groups as judged by direct ELISA (Fig. 2A). However, total SOD activity increased significantly (*p* = 0.04) (Fig. 2B), while total GPX activity was decreased in the AD-STG compared to controls (*p *= 0.02) (Fig. 2C). This data was consistent with an imbalance in H_2_O_2_ producing *vs* consuming enzymatic activity and warranted further investigation to assess other endogenous H_2_O_2_-consuming enzymes.

**Figure 3:**
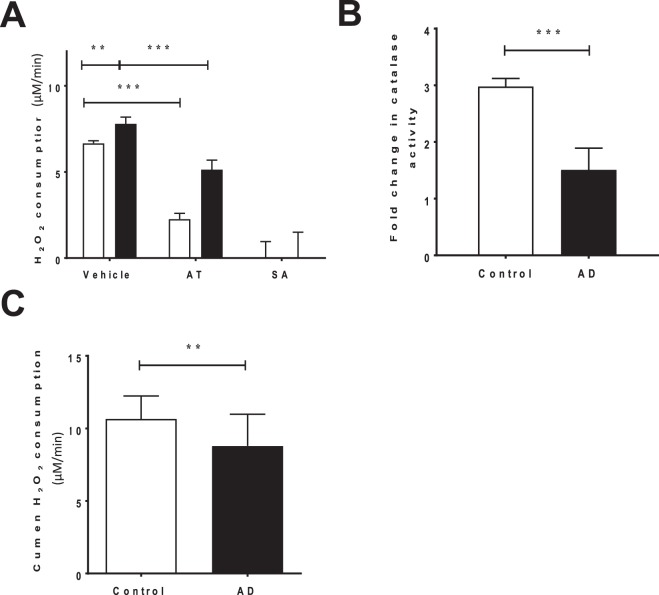
Determinations of H_2_O_2_-consuming activity in the AD- and control-STG. (**A**) Total H_2_O_2_ consuming activity in the control and AD STG was assessed by treating supernatants with 0.1% v/v H_2_O_2_ (vehicle). Additionally, H_2_O_2_ consuming activity was assessed following pre-treatment with the catalase inhibitor, aminotriazole (AT) and sodium azide in both subject groups. (**B**) H_2_O_2_ consumption attributed to catalase (difference between vehicle and AT consumption rates) displayed as a fold change relative to the control group (n = 5 controls and n = 5 AD subjects) (**C**) A modified FOX assay displaying the peroxiredoxin specific consumption of cumen H_2_O_2_ (n = 19 control and n = 19 individual AD cases). Data represent mean ± SD; *p < 0.05, **p < 0.01 & ***p < 0.0001.

Surprisingly, the capacity for the AD-STG to degrade exogenously added peroxides was slightly greater than control-STG (*p* = 0.002), as judged by an *ex vivo* assays measuring rates of H_2_O_2_ consumption (Fig. 3A). To understand the specific contribution of CAT to this peroxide consuming activity in control and AD brains, H_2_O_2_ decomposition by added STG-homogenate was reassessed in the presence of aminotriazole (AT), a CAT-inhibitor. Under these conditions, H_2_O_2_ consumption diminished more in the control group than in the AD cohort, suggesting a higher level of catalase specific activity in control STG (*i.e*., compared the difference in activity between the white bars (control) and then the black bars (AD) in the absence and presence of AT, Fig. 3A). Overall, we determined a 1.5-fold lower CAT activity in the AD-STG than the corresponding enzymatic activity in the control-STG (p < 0.001) (Fig. 3B). This decreased CAT activity exacerbates the documented loss of GPX activity in the same STG tissues (Fig. 2C) and together imply that the AD-STG shows a limited ability to enzymatically decompose accumulating H_2_O_2_.

Overall, total consumption of H_2_O_2_ is likely to be mostly ascribed to a combination of CAT and heme-peroxidase activity in the clarified brain homogenates as sodium azide (SA; a highly active heme-peroxidase inhibitor)^20^, completely inhibited peroxide consumption in both the control and AD cohorts (Fig. 3A). Note, although azide is a potent inhibitor of catalase activity, azide-insensitive catalase activity has been reported in bacteria^21^, and this may impact on the interpretation of these inhibition studies.

The peroxiredoxin (Prx) family of anti-oxidant enzymes, are also a pathway for eliminating H_2_O_2_, and have also been implicated in the pathogenesis of AD^22^. Therefore, we measured total peroxiredoxin activity by assessing the selective consumption of cumene-hydroperoxide. Similar to catalase and total GPX activity, peroxiredoxin activity decreased slightly (and significantly) (*p* = 0.004) in the AD-STG compared to the controls (Fig. 3C). Together these data indicate that the AD-STG is less capable of detoxifying accumulating (low molecular weight) peroxides via multiple antioxidant enzymic pathways compared with the corresponding control tissue.

**Figure 4:**
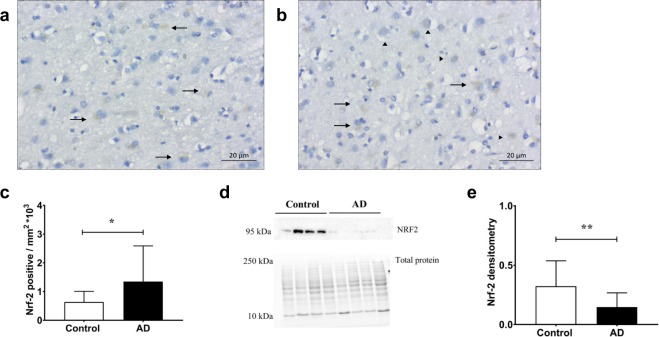
NRF-2 protein expression and distribution in the AD- and control-STG. Photomicrographs of NRF2 protein staining of the STG grey matter in a (**A**) control and (**B**) AD case. (**C**) Image semi-quantification of NRF2 positively stained pixels. Data represent mean ± SD. Within image panels → indicates cytosolic staining and ►  indicates nuclear staining, n = 14 for control and n = 16 individual AD subjects. (**D**) Representative blots for cystosolic fraction from control and AD samples (23 μg protein loading) displaying NRF2 band (95 kDa, refer to Supplementary Fig. S2 for complete gel image; protein band at ~50 kDA was not included in the assessment) and corresponding total protein bands imaged prior to protein transfer. (**E**) Densitometry of NRF-2 immunoblot bands using cytosolic STG fractions, normalized to total protein. Note, multiple blots were processed in parallel with a constant exposure time (125 sec) to allow semi-quantitiation over the complete control and AD sample sets. Data represent mean ± SD, n = 19 controls and n = 19 individual AD cases. *p < 0.05 & **p < 0.01.

Additionally, total and cytosolic NRF-2 protein expression was assessed using a combination of immunohistochemistry and western blotting respectively. Consistent with the gene response, a statistically significant increase in total *NFE2L2* expression was observed in the AD cohort (*p* = 0.05) (Fig. 4A–C). To assess potential differences in NRF-2 subcellular localisation, NRF-2 protein levels were also assessed in cytosolic homogenate fractions. A recent consensus in the literature indicates that the molecular weight for NRF-2 occurs between 95–100 kDa^23^ therefore, bands detected at mass 95 kDa were used quantify a significant decrease in cytosolic NRF-2 levels in the AD cohort (*p* = 0.004) (Fig. 4D and E, refer to Supplementary Fig. S2), suggesting that NRF-2 levels are inherently lower in the AD-STG and/or that NRF-2 may be sequestered to the nucleus in this cohort.

**Figure 5:**
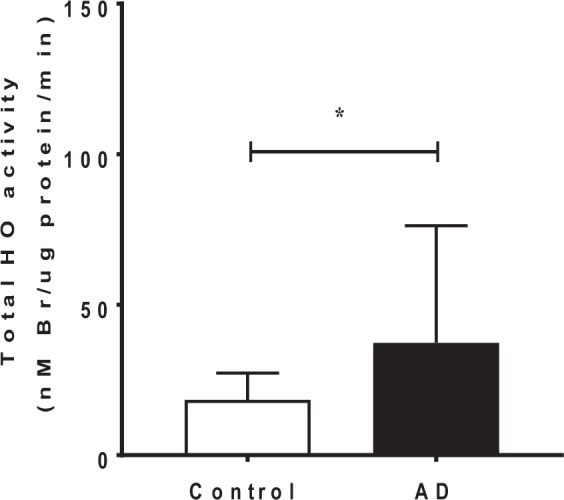
Total HO activity in AD and control STG. Microsomal fractions of STG homogenates exposed to hemin and biliverdin reductase allowed for the HO dependent conversion of heme-to-bilirubin. Data reflect bilirubin levels, as determined by HPLC analysis. Data represent mean ± SD, n = 19 for control and n = 21 individual AD cases. *p < 0.05.

Total HO activity, a downstream transcriptional target of NRF-2, was also assessed in the homogenized brain tissues. Overall, we determined a significant increase in total HO activity, as indicated by increased bilirubin production in the *ex vivo* assay (*p* = 0.04) (Fig. 5; see Supplementary Fig. S3 for exemplar chromatograms). This result taken together with the corresponding gene studies that showed a selective increase in the inducible *HMOX1* mRNA but not *HMOX2* mRNA, suggest that active HO-1 protein is expressed in the AD-STG and this likely accounts for the parallel increase in total HO activity in these same tissues.

### Assessing oxidative damage in the AD-STG

**Figure 6:**
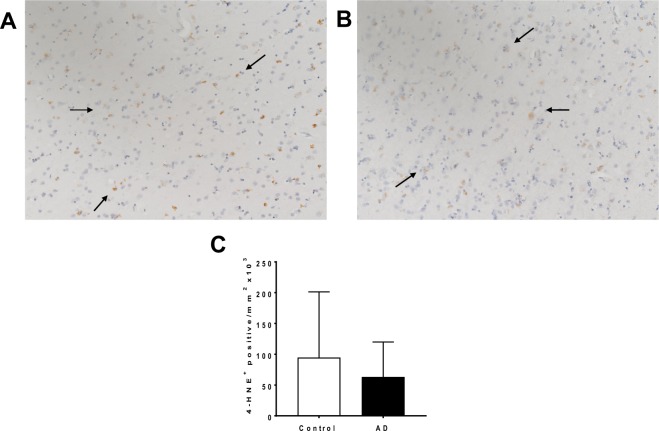
4-HNE immune-reactivity in AD- and control-STG. Photomicrographs of 4-HNE immunostaining of the STG grey matter in a (**A**) control and (**B**) AD case (**C**) Image quantification of 4-HNE^+^ stained pixels. Data represent mean ± SD, n = 19 for control and n = 19 AD cases.

Oxidative tissue damage was determined by immunostaining for 4-hydroxynonenal (4-HNE)^+^. There were no difference between cases and controls (Fig. 6A,B) suggesting that despite the documented decrease in antioxidant enzymes responsible for detoxifying H_2_O_2_, oxidative protein damage remained unchanged. Semi-quantitation of the 4-HNE^+^ staining confirmed no difference in levels of this biomarker of oxidation (Fig. 6C). While this specific form of oxidized damage was unaffected, the altered patterns of anti-oxidant response elements documented in the AD-STG are consistent with these tissues experiencing a subtle heightened oxidative stress.

### Caspase-dependent apoptosis

**Figure 7:**
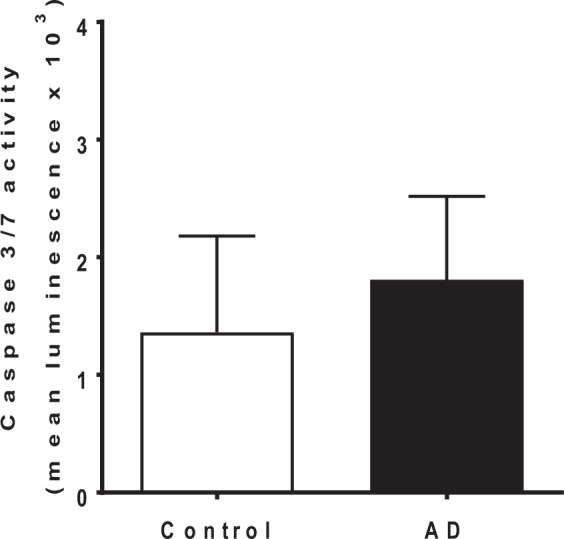
Caspase 3/7 activity in AD and control-STG. Supernatants were treated using the Caspase-Glo 3/7 assay kit (Promega). Caspase 3/7 activity was determined for each homogenate after 60 min of monitoring the readout. Data represent mean ± SD, n = 19 for control and n = 20 individual AD cases.

To investigate whether changes in H_2_O_2_ in the AD-STG promoted pro-apoptotic cell death pathways, caspase 3/7 activity was determined in control- and AD-STG homogenates. This biomarker of apoptosis was marginally higher in the AD-STG homogenates relative to the controls, although this did not reach statistical significance (*p* = 0.08) (Fig. 7). Therefore, our data are consistent with unaltered capsase-mediated cell death (*i.e*., intrinsic apoptosis) in the AD-STG.

### Correlations between biochemical markers and AD pathology

**Figure 8:**
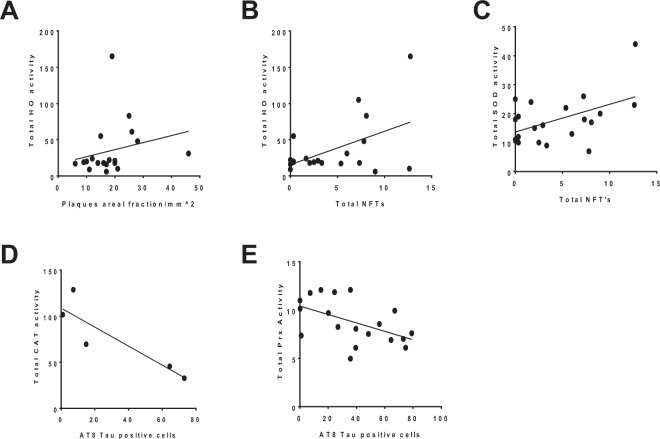
Correlational outcomes of HO, SOD, CAT and Prx activity with AD pathology. Total HO activity in the AD STG was correlated with both (**A**) plaque areal fraction (*p* = 0.04, r^2^ = 0.21) and (**B**) total NTFs (*p* = 0.03, r^2^ = 0.24) In addition, (**C**) total NFTs were correlated with total SOD activity (*p* = 0.03, r^2^ = 0.23) while the number of AT8- tau positive cells were correlated with (**D**) total CAT activity (*p* = 0.05, r^2^ = 0.77) and (**E**) total Prx activity (*p* = 0.02, r^2^ = 0.26).

Overall there was a paucity of correlations identified and some inconsistency in the relationships using different biomarkers of AD. From those that showed linear relationships total HO activity was significantly and positively correlated with STG plaque areal fraction (p = 0.04, r^2^ = 0.21) (Fig. 8A) as well as total NFTs in the AD-STG (r^2^ = 0.24, *p* = 0.03) (Fig. 8B). In addition, total SOD activity was positively correlated with total NFTs (r^2^ = 0.23, *p* = 0.03) (Fig. 8C), while total CAT (Fig. 8D) and Prx (*p* = 0.02, r^2^ = 0.26) (Fig. 8E) activities were negatively correlated with AT8-tau positive cells.

Interestingly no relationships between the areal fraction of plaques and any other antioxidant protein activities were identified in the brain tissues analysed here. Nor were plaques related to cytosolic or total NRF-2 expression (see Supplemental Fig. S4). Also, we found no significant correlations between AT8-tau and total NFT immunoreactivity or either of these pathological tau indices and biochemical activity of the antioxidant enzymes (Supplemental Figs S5 and S6 respectively).

## Discussion

Despite its inherent retrospective nature post-mortem brain tissue remains the primary vehicle for mechanistic investigations in humans. We have previously suggested that investigating the less affected areas of the AD brain may represent a surrogate for more severely affected areas at an earlier stage in the pathogenesis of AD^24^. Albeit it is also possible that the STG is resistant to AD rather than belatedly affected. In an effort to understand early molecular events associated AD we investigated the STG, a region that is moderately affected in the AD brain with characteristic Aβ pathology, including neuritic plaques, but only mild tau-related changes and minor neuronal loss.

The popular ACH states that a build-up of soluble Aβ oligomers in the brain parenchyma triggers a sequence of neurotoxic events, including oxidative stress that eventually leads to NFT formation and neuronal death^25^. An alternative hypothesis has been proposed that places Aβ accumulation secondary to oxidative stress^26^. Here we specifically hypothesized that examining oxidative stress in a belatedly affected area of the AD brain (as evidenced primarily by Aβ accumulation) would allow us to help clarify the temporal relationship of oxidative stress in AD pathogenesis. The majority of our findings appear to support an increase in oxidative stress with plaques being associated with subtle changes in redox balance insufficient to result oxidative lipid/protein damage.

Attenuation of the antioxidant actions of peroxide-consuming enzymes CAT, GPX and peroxiredoxins with a parallel increase in SOD activity (responsible for converting the superoxide radical anion to H_2_O_2_) likely creates an intracellular milieu containing excess peroxides in the AD-STG. Although we were able to show decreases in specific peroxidase and catalase activity, the total H_2_O_2_ consumption measured in STG clarified homegenates was initially higher in AD-STG. We employed a strategy using specific inhibitors or peroxide substrates to evaluate contributions of GPX, CAT and peroxiredxin activities, which were all determined to be lower in the AD-STG. Thus, enhanced dismutation of superoxide radical anion leads to elevated H_2_O_2_ that is ineffectively degraded by virtue of decreased capacity for the STG to consume this pro-oxidant. That the total H_2_O_2_ consumption was initially higher in the AD-STG than in control-STG requires further discussion. This apparent paradox may reflect methodological limitations or that H_2_O_2_ is consumed by reactions with unbound metal ions released through tissue processing. Others have demonstrated that transition metals increase around plaques and NFTs in the AD brain^27^. These transition metals may contribute to the consumption of H_2_O_2_ to yield other ROS species^28^ rather than being degraded to benign products. For example, Fe^2+^ and H_2_O_2_ yield the highly reactive ROS, OH^•^ via the Fenton or Haber–Weiss reactions^15^. However, our data indicating that sodium azide treatment inhibited the majority of *ex vivo* H_2_O_2_ consumption, strongly suggesting that free metals play only a minor role in this process, with a majority of transition metals likely to remain bound to proteins and unable to participate in unregulated redox reactions. Furthermore, the increased consumption of exogenous peroxide is unlikely to reflect a methodological limitation as the outcome from the studies with the heme-poison sodium azide points to other pathways of *ex vivo* H_2_O_2_ consumption linked to other peroxidases that are present in brain tissues and not identified here.

A study by Gsell and colleagues provides further evidence for an accumulation of H_2_O_2_ in the AD brain, as judged by assessments of the ratio of SOD/CAT activity. This study reported that SOD activity remained unchanged in the AD brain, however similar to outcomes we document here, a decreased CAT activity yielded an overall increase in oxidative stress through an accumulation of H_2_O_2_. These results were consistent in multiple post-mortem brain regions, ranging from mild to severely affected regions of AD^29^ and we have built on this outcome by demonstrating decreased activity for other enzymes responsible for H_2_O_2_ consumption in the AD brain. Another study reports decreased mitochondrial SOD 2 levels and unchanged SOD1 levels in patient serum, relative to age and gender matched controls^30^. While any deviation from age and gender matched controls may still reflect altered redox homeostasis in AD, differences in SOD levels reported in our present study may reflect difference in redox states between the peripheral and central nervous system of early stage AD patients. Notably, small sample size, has been suggested to contribute to the inconsistent SOD serum levels that have been reported in the literature (reviewed by Chang *et al*.^31^). Thus, Chang and colleagues identified that the three studies with over 80 cases all report significantly increased SOD levels. Moreover, differences in SOD levels may reflect different disease stages. Consistent with our biochemical outcomes measured in the STG, which reflects the early changes in AD etiology, comparisons of leukocyte SOD levels in patients with severe and mild AD vs corresponding control subjects suggest that SOD levels are elevated in the early stages of AD, however appear to be depleted in the later stages of disease progression^32^. Although our data suggests a likely accumulation of H_2_O_2_ in the AD-STG, we found no evidence of increased ROS-mediated damage at least in the form of 4-HNE^+^ immuno-staining. As previous reports indicate that differences in tissue-storage time influence immuno-staining intensities^33^, and so may impact on the interpretation of this immune-staining approaches, 4-HNE^+^ immuno-staining was repeated with a sub-cohort that were more closely matched for storage time; no difference in result was obtained. Despite no change in 4-HNE^+^, we chose to assess additional oxidative markers to corroborate this outcome and demonstrate oxidative stress in the AD-STG.

The transcription factor NRF-2 is now considered a master redox switch that initiates a suite of cyto-protective genes that are central to cell survival^34^. Both gene and possibly nuclear localisation of NRF-2 were increased in the AD-STG, suggestive of stress-induced NRF-2 activation early in AD pathogenesis. By contrast, studies using highly affected areas of the AD brain suggest that nuclear levels, and thus activation, of NRF-2 decrease in the AD brain^35,36^. This suggests that NRF-2 protein levels may decrease with increasing severity of AD pathology. Overall, our data together with outcomes from others^37,38^ provide further evidence to suggest that therapies targeting an enhancement of NRF-2 dependent pathways may serve as a promising treatment for pre-symptomatic AD subjects.

Here we found an increase in expression of inducible HO-1 protein; a downstream target of transcriptional NRF-2. As levels of *HMOX-2* mRNA remained unchanged the simplest explanation for the observed increase in total HO activity, measured in an *ex vivo* assay employing homogenates from AD-STG, is likely attributed solely to alteration in the inducible HO-1 protein. Although previous studies using AD hippocampal tissues also reported increased HO-1 expression, this has not translated to increased activity or neuroprotection^39^.

While our data suggest that total HO activity may be enhanced and protective early in the development of AD pathology, it must be recognized that our assessment of HO activity was determined using an *ex vivo* assay that added liver-derived biliverdin reductase to convert biliverdin into bilirubin, with the latter biomarker of HO activity detected by liquid chromatography. To add biological context to our result, recent studies have indicated decreases in biliverdin reductase activity with increasing severity of AD pathology^40,41^ thereby potentially limiting the conversion of biliverdin to the more potent antioxidant bilirubin. Limitations in the conversion of biliverdin to bilirubin may therefore be an important feature of whether HO-1 confers neuro-protection in different individuals, and in turn, potential resistance to developing advanced AD pathology.

Stocker and colleagues^42,43^ have demonstrated soluble bilirubin to be a potent inhibitor of lipid peroxidation. Also, inhibition of HO-1 activity in stress induced endothelial cells has demonstrated increased sensitivity to ROS mediated damage, with restored effects of cell viability attributed to the addition of bilirubin^44^. A gene deletion of biliverdin reductase has further been associated with increased endogenous oxidative stress^45^, suggestive of a direct role for bilirubin in conferring protection by HO-1, albeit that carbon monoxide, another by-product of the catabolic heme pathway can also confer neuroprotection^46^.

Our data suggests a requirement for a functional HO/BV/BR system and that augmenting this pathway in AD may be a target for future therapy. However, while the NRF-2/HO-1 pathway may seem an attractive therapeutic target for AD^47^ a comprehensive understanding of mechanisms regulating HO-1 expression in brain cells, whether other enzymes such as biliverdin reductase are active, and how these mechanisms link to neuropathological changes is essential before this pathway can be exploited for therapeutic development. Consistent with this conclusion, studies with a transgenic AD mouse model demonstrate^48,49^ increased levels of oxidative stress, AD pathology and enhanced cognitive dysfunction when double crossed with a knockout of antioxidant defences, including deficiencies in SOD^50,51^, and GPX^52^, while overexpression of SOD appears to reduce ROS levels, memory deficits and plaque load in respective AD mice^48,49^. Similarly, inducing NRF-2 activity in transgenic AD mice yields enhances neuroprotection against oxidative stress attributed to increased HO-1 expression^53,54^.

On balance the majority of data obtained here suggest that oxidative stress is subtly elevated in the AD-STG, and potentially in the early stages of AD pathogenesis, as judged by altered activity of a number of antioxidant response elements albeit in the absence of detectable oxidative protein damage. The limited extent of pathology and largely preserved complement of neurons observed in the STG suggests that a degree of resistance to oxidative stress is operative. Our findings are consistent with a mechanism whereby the STG, relative to more severely affected areas in the medial temporal lobe, retains the ability to dismutate the superoxide radical anion into H_2_O_2_, stimulating NRF-2 to increase total HO-1 and perhaps BVR activity to ultimately resist neuronal loss that is characteristic of AD.

## Methods

### Case selection

As previously described^19^ frozen and fixed specimens of superior temporal gyrus (STG) from 21 age, gender- and APOE ε4-matched AD cases and 19 neurologically normal controls were obtained from New South Wales Brain Banks (NSWBB) following approval by the University of Sydney’s Human Research Ethics Committee (#HREC 11245/USYD 12734) and the NSW Scientific Advisory Committee. Pathological AD was diagnosed by a neuropathologist using standard diagnostic criteria^55^ and cases were either Braak stage IV (n = 1), V (n = 4) or VI (n = 12); four cases did not fit staging criteria (see Supplementary Table S1). Brains were from subjects that were to the best of our knowledge unaffected by any infectious disease, or other known neurological disorders. Any brain sample obtained with post-mortem intervals (PMI) > 2.6 days or significant agonal events were excluded from this study. Control brain tissue employed here was derived from a cohort of coronial cases, with the primary mode of death recorded as myocardial infarction.

### Tissue preparation

Samples of frozen brain tissue (30 mg) were suspended in 600 μL lysis buffer containing 6 μL beta-mercaptoethanol as provided by the Bioline Isolate II RNA Kit (Bioline, Australia; BIO-5207) then homogenized with a rotating piston arrangement (Wheaton Specialty Glass, NJ, USA) at 500 r.p.m.^56^, and centrifuged (5 min, 13,000 × *g*) prior to RNA extraction. Total RNA was extracted according to the manufacturer’s instructions then quantified using a Nanodrop ND-1000 spectrophotometer (Thermo Scientific, Wilmington, USA); RNA quality was determined using a BioAnalyzer 2100 (Agilent Technologies, Santa Clara, USA) and individual RIN values shown in Supplementary Table S1. All samples were stored at −80 °C prior to use.

Additional samples of frozen human brain STG tissue (~100 mg) were cut into small pieces, suspended in 2 mL Buffer (50 mM PBS, pH 7.4) containing: 1 mM EDTA, 10 μM butylated hydroxytoluene and a Protease Inhibitor Cocktail tablet (Roche Diagnostics, Bern Switzerland; 1 tablet/50 mL buffer). The suspension was homogenized as described above and the homogenates divided into 0.2 mL aliquots, immediately snap frozen in liquid nitrogen and stored at −80 °C. As required, homogenates were thawed at 20 °C then centrifuged (5 min, 13,000 × *g*) to clarify the supernatant for use in all biochemical and immune-assays (see below). Protein concentrations for all samples were determined using the Bicinchoninic Acid (BCA) assay (Sigma-Aldrich, St Louis, USA) and average values used to normalize all subsequent biochemical parameters measured.

### Materials

Chemicals were obtained from Sigma-Aldrich (Castle Hill NSW 1765, Australia), unless stated otherwise. All solutions were freshly prepared using MilliQ® Water or high quality analytical grade organic solvents and, where appropriate, sterilized prior to use.

### Gene expression

Complementary DNA (cDNA) was synthesized using the Tetro cDNA Synthesis Kit (Bioline, Australia; BIO-65043) and gene expression was determined using RT-qPCR performed with a Roche LightCycler 480 system (Roche Diagnostics Corporation, Indianapolis, Indiana, USA) according to MIQE recommendations^57^ as previously described^19^. Primer efficiencies and sequence pairs used for all RT-qPCR analyses are shown in Table 2. Melt curves were generated using the LightCycler 480SW *v*1.5.1 software to demonstrate a single PCR product (see Supplementary Fig. S7). RT-qPCR analysis was carried out using the GenEx qPCR data analysis software (*v*5.3.2) (MultiD Analyses, Goteborg, Sweden). Importantly, correlations were performed to assess the impact of RNA integrity number (RIN) and tissue pH on expression levels of the genes of interest studied here as described in detail elsewhere^19^. This approach afforded the selection of a suitable reference gene that normalized RIN/pH differences between case and control tissue samples.

**Table 2:** Gene specific primer sequences and annealing temperatures for RT-qPCR.

Gene	Primer Sequence	Annealing Temp (°C)	Primer efficiency value
*SDHA*	(F) – TGGGAACAAGAGGGCATCTG (R) – CCACCACTGCATCAAATTCATG	65	2.10
*NFE2L2 (e1-2)*	(F) – CGTCCCAGCAGGACATGG (R) – GCTCATACTCTTTCCGTCGC	65	2.00
*HMOX1*	(F) – ACTGCGTTCCTGCTCAACAT (R) – GGGGCAGAATCTTGCACTTT	65	2.13
*HMOX2*	(F) – CACTGGCCAGAGAGACCTTG (R) – CTCCAGGGCACCTTTCTCTT	65	2.13
*SOD1*	(F) – GGTGTGGCCGATGTGTCTAT (R) – CACCTTTGCCCAAGTCATCT	65	2.08

Primers were synthesized by Geneworks (Hindmarsh, South Australia) and stored at −20 °C. Prior to use the stock solutions were diluted 10x to yield a working stock solution (10 μM). Primers sequences were blast searched and confirmed to target the gene of interest in these human tissues.

### Immunohistochemistry

Briefly, formalin-fixed, paraffin embedded (FFPE) sections were obtained from the contralateral STG of all individuals. FFPE-STG sections (thickness 10 μm) were stained with cresyl violet and Garvey-modified silver stain or immunostained for phospho-tau (AT8, mouse monoclonal, Thermo Scientific, MI161393)^19^.

In addition, FFPE-STG sections were immunostained for 4-hydroxy-2-nonenal (4-HNE^+^) and NRF-2. Briefly, sections were rehydrated and antigen retrieved using a decloaking chamber (Biocare Medical, CA, USA). Samples were heat retrieved at 95 °C for 30 min in a pH 9 buffer (Agilent Technologies, Santa Clara, CA, USA, #S2367), cooled to 23 °C and treated with 3% v/v H_2_O_2_ in dH_2_O to block for endogenous peroxidase activity. Sections were then incubated with a serum-free protein block (Agilent Technologies, #X0909) for 30 min, followed by application of primary antibody including: a polyclonal anti-4-HNE antibody (1:200 v/v; overnight incubation at 4 °C) (Bioss, Boston, USA, #bs-6313R) and an oligoclonal anti-NRF-2 antibody (1:200 v/v, 1 h incubation at 23 °C) (Thermo Fisher Scientific, MA, USA, #710574). Products were then visualized via incubation with the DAKO EnVision™ + System-HRP (Agilent Technologies, #K4002; 30 min at 23 °C), followed by a 5 min incubation with DAB (Agilent Technologies, #K3468). Sections were counterstained using hematoxylin, dehydrated and mounted with DPx.

The oxidation biomarker 4-HNE was selected as a measure for tissue oxidation status as critically reviewed by Esterbauer and colleagues^58^. The 4-HNE immuno-sections were imaged simultaneously using the same parameters with an Axio Scan.Z1 slide-scanning microscope (Carl Zeiss, Jena, Germany). Entire sections were scanned using a 10x objective and imported into MetaMorph software program (v7.6 Universal Imaging Corp., Downington, Pa, USA). The lasso tool was used to outline the grey matter (GM) and white matter (WM) for each section and the threshold tool used to calculate the percentage of area stained in the GM and WM of each case.

Where required NRF-2 immunoreactivity was imaged using an Axio Lab.A1 microscope (Carl Zeiss, Jena, Germany). Sections were imaged using a 40x objective, and converted to JPEG files for analysis via the ZEN software (Carl Zeiss, Jena, Germany). Pixel counts were then determined using ImageJ software. The average percentage of area stained in the GM of each section was used for statistical analysis.

### Western blot analysis

Western blots were performed on tissue homogenates as described previously^59^. Clarified supernatants were, diluted to matching protein concentration (23 μg total) and heated with Laemmli loading buffer (BioRad, Hercules, CA; 95 °C, 5 min) and separated on a 12% TGX gel (Bio-Rad, #161-0180) by SDS-PAGE. Prior to protein transfer to the blotting membrane all protein bands were visualized using a ChemiDoc^TM^ MP system (BioRad) and total band densities were obtained via Image Lab *v*5.2. Proteins were then transferred onto a PVDF membrane, blocked in 5% w/v skim milk powder (in 0.1% v/v TBS-T; 1 h, at 23 °C), then incubated with a mouse anti-Phospho-Tau (AT8; Thermo Scientific, Waltham, MA, #MN1020) overnight at 4 °C. Membranes were blocked at 4 °C and incubated with a rabbit anti-NRF2 antibody (Thermo Fisher Scientific, #710574) for 3 h, at 23 °C and then incubated with either an anti-mouse or anti-rabbit HRP-conjugated secondary antibody (dilution 1:5000 v/v; 1 h at 23 °C). Protein bands were visualized using Luminata Forte HRP substrate (Millipore, Billercia, USA) and imaged on a ChemiDoc^TM^ MP system (BioRad).

### Direct Enzyme-Linked Immunosorbent Assay (ELISA)

Clarified supernatants (15 µg protein/mL; 100 µL) were prepared in coating buffer (containing 15 mM NaCO_3_ and 35 mM NaHCO_3_, pH 7.4), loaded onto a Maxisorp plate (Nalge Nunc, Rochester, USA) and incubated overnight at 4 °C. The plate was then blocked for 2 h in 1% w/v skim milk and incubated with primary antibody (3 h, 23 °C). Primary antibodies included goat anti-SOD1 (Santa Cruz, USA, #sc8637; dilution 1:200 v/v), goat anti-SOD2 (Sigma, USA, #S2147; 1:200 v/v) and goat anti-GPX1 (R&D Systems, USA, #AF3798; 1:500 v/v). Wells were then incubated with secondary antibody (1:5000 v/v dilution) (anti-mouse IgG-HRP and anti-rabbit IgG-HRP; 1 h at 20 °C). Next, a solution of ABTS substrate (Life Technologies, Carlsbad, CA) was added to each well followed by (1% w/v SDS) and monitoring at 410 nm using the FLUOstar Omega reader with values normalized to total protein.

### Antioxidant activity assays

#### Total Superoxide dismutase (SOD) activity

The combined SOD1/2 (or total SOD) activity^60^ was assessed in samples of clarified supernatants (20 µL volume loaded) by measuring the inhibition of pyrogallol auto-oxidation monitored at 405 nm at 5 min intervals over 1 h of monitoring to establish a linear rate of superoxide radical anion evolution. Total SOD activity was expressed as a relative rate per min and normalized to the total protein level in the corresponding samples.

#### Total Glutathione peroxidase (GPX) activity

Assessment of total GPX activity was based on the GPX-dependent consumption of NADPH monitored at 340 nm^61^. Clarified supernatant (10 µL) were treated with a reaction mixture containing: 1 mM EDTA, 1 mM sodium azide, 1 mM glutathione, 1 U glutathione reductase, 0.25 mM NADPH and 50 mM phosphate-buffered saline (PBS) (volume 125 µL). A second mixture containing 350 µM H_2_O_2_ in 50 mM PBS (volume 65 µL) was then added to each well to a final volume of 200 µL. Absorbance was read at 5 min intervals over a 1 h period. Total GPX activity was expressed as a relative rate per min; all data was normalized to total protein in each sample.

#### Total Catalase (CAT) activity

To assess CAT activity, clarified supernatants (3.5 µL volume loaded) were treated with either 20 mM aminotriazole (AT) (a dose that inhibits CAT activity^62,63^, the heme-poison sodium azide (SA) (20 mM) as an additional negative control or 50 mM PBS (as vehicle control) for 5 min at 20 °C. Next, an aliquot (95 µL) of 0.1% v/v H_2_O_2_ was added and absorbance was read at 240 nm using a FLUOstar Omega reader at 5 min intervals over 2 h. The rate of H_2_O_2_ decomposition was assessed by the gradient of the linear data analysis (coefficient R^2^ ranging 0.9–0.95) and normalized to total protein in the corresponding samples with CAT activity ascribed specifically to an AT-inhibited fraction of peroxide consumption.

#### Total Peroxiredoxin (Prx) activity

A modified ferrous oxidation-xylenol orange (FOX) assay was used to assess total Prx activity as described previously^64^. Cumene hydroperoxide was selected to enhance specificity in this enzyme activity assay as Prx activity shows enhanced catalytic efficacies and specificities toward tertiary lipid hydroperoxides^65^. Samples of clarified supernatants (3.5 µL) were incubated with 100 µM cumene hydroperoxide and 100 µM dithiothreitol (final volume of 10 µL) for 5 min at 20 °C. An aliquot of FOX solution (250 µM ammonium sulfate, 100 mM D-sorbitol and 125 µM xylenol orange) was then added to yield a total volume of 100 µL and the mixture was incubated at 20 °C. After 30 min, absorbance was read at 560 nm using a FLUOstar Omega reader and data was normalized to total protein.

#### Total hemeoxygenase (HO) activity

Clarified supernatants from brain homogenates (500 μL) were subject to ultracentrifigation (100 000 × *g*; 1 h at 4 °C). The resulting microsomal pellets were resuspended in 100 µL PBS and used to determine total heme-oxygenase (HO 1/2) protein activity. For total HO activity microsomes were mixed at a final ratio f 2:1 (brain homogenate-microsomes/isolated at liver-microsomes) in 100 µL buffer (250 mM sucrose, 20 M Tris, pH 7.4). Next, the microsomal mixtures were treated with 1 mM NADPH, 2 mM D-glucose-6-phosphate (G-6-P), 1U glucose-6-phosphate dehydrogenase and 1 µL of 2.5 mmol/L hemin and incubated at 37 °C in the dark. After 1 h, the reaction was stopped by addition of 100 µL ethanol/DMSO (95:5 v/v). The mixture was then centrifuged at 13000 × *g* for 5 min, and the supernatant was assayed for bilirubin with liquid chromatography^20^. Finally, total HO activity was determined as the protein normalized level of bilirubin.

### Caspase activation

Caspase-3 and -7 activities were assessed using a commercial kit (Caspase-Glo^TM^ 3/7, Promega, Fitchburg, USA) as per the manufacturer’s instructions. Briefly clarified supernatants (50 μL) were diluted according to individual protein concentrations to yield final protein concentrations of 0.5 mg protein/mL. These protein-adjusted samples were then added to Caspase-Glo^TM^ 3/7 reagent (1:1 v/v, 20 °C for 60 min) and luminescence measured using a FLUOstar Omega reader (BMG LABTECH, Offenburg, Germany). Total caspase 3/7 activity was represented as mean luminescence after 60 min of monitoring.

### Statistical analysis

All data are presented as the mean ± SD. Statistical tests including two-tailed t-tests (for paired data sets), and ANOVA were performed with GraphPad Prism (*v*6); *p* ≤ 0.05 was regarded as significant.

### Data availability statement

The datasets generated during and/or analysed during the current study are available from the corresponding author on reasonable request. All data generated or analysed during this study are included in this published article (and its Supplementary Information files).

## Electronic supplementary material

Supplementary data
