# Supplementary material for: Evidence supporting oxidative stress in a moderately affected area of the brain in Alzheimer’s disease
Source: Sci Rep. 2018 Aug 1;8:11553. doi: 10.1038/s41598-018-29770-3 (PMC6070512; doi:10.1038/s41598-018-29770-3)
Supplement: Supplementary file 1 — Supplementary data [file 41598_2018_29770_MOESM1_ESM.docx]

**Evidence supporting oxidative stress in a moderately affected area of the brain in Alzheimer’s** **disease**

Priscilla Youssef,^1^, Belal Chami,^1^ Julia Lim,^2^ Terry Middleton,^2^, Greg Sutherland,^2^ and Paul K. Witting ^1*^

^1^Redox Biology Group, Discipline of Pathology, University of Sydney, Sydney, NSW, 2006, Australia

^2^Neuropathology Group, Discipline of Pathology, The University of Sydney, Sydney, NSW 2006, Australia

* Correspondence should be addressed to P.K.W (email paul.witting@sydney.edu.au)

**Supplementary data**


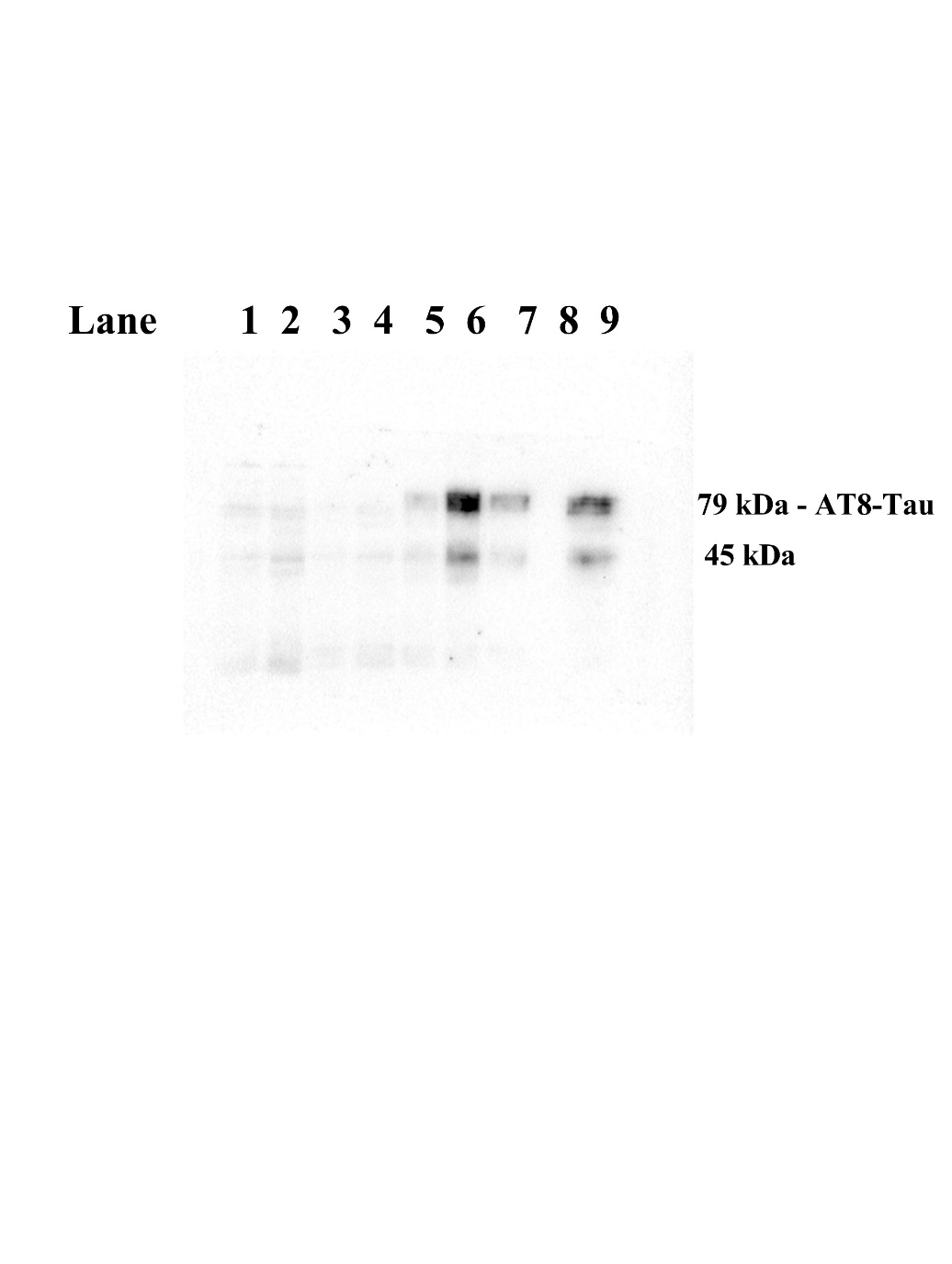


**Supplementary Figure S1**. Full length western blot probed for AT8-tau. Blot displays sample supernatants for age and gender matched control (Lanes 1-4) and AD (lanes 5-7, 9) patients. Blot taken at 41.1 sec exposure time.


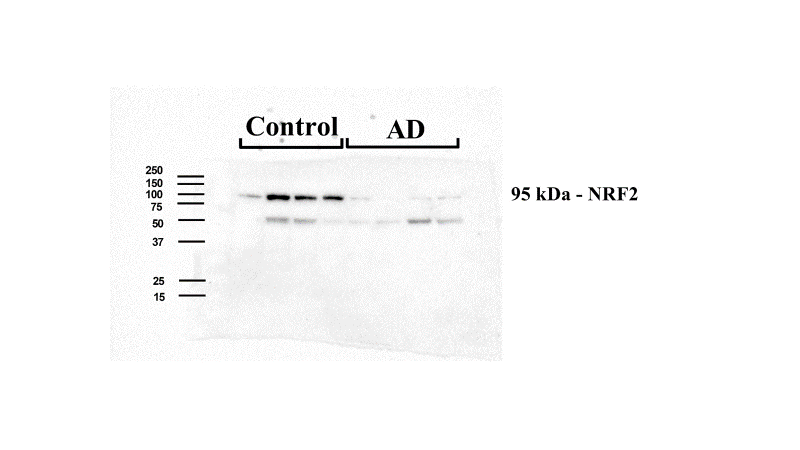


**Supplementary Figure S2**. Full length western blots of supernatants displaying age and gender matched control and AD samples probed for NRF2 (image at 125 sec exposure).


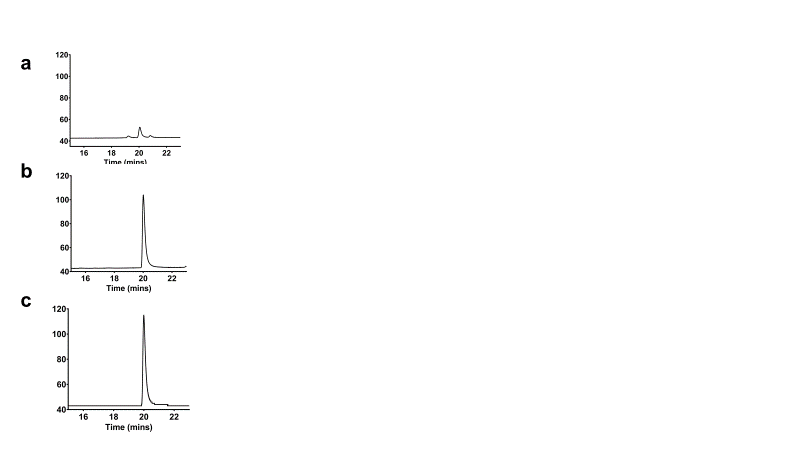


**Supplementary Figure S3**. Representative HPLC chromatograms displaying bilirubin production in AD and control STG using methods described in the corresponding main paper. (A) Bilirubin standard displayed 3 peaks, with the central isomer peak eluting at ~20 min. Panels (B) and (C) show representative chromatograms from an AD and control STG samples respectively.

**Supplementary Figure S4**. Plaque areal fraction correlated poorly with (A) total SOD, (B) GPx, (C), CAT and (D) Prx activity; data assessed using Prism *v*7.


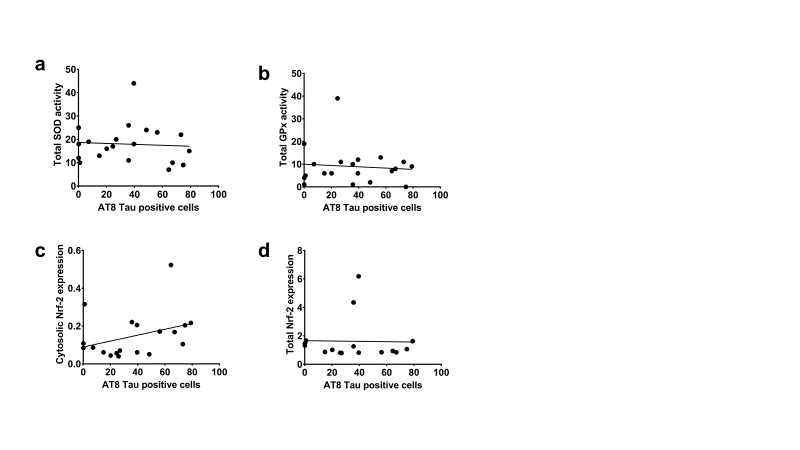


**Supplementary Figure S5**. AT8 positive neurons correlated poorly with (A) total SOD (B) GPx, activity (C) cytosolic NRF-2 protein expression or (D) total NRF-2 expression; data assessed using Prism *v*7.


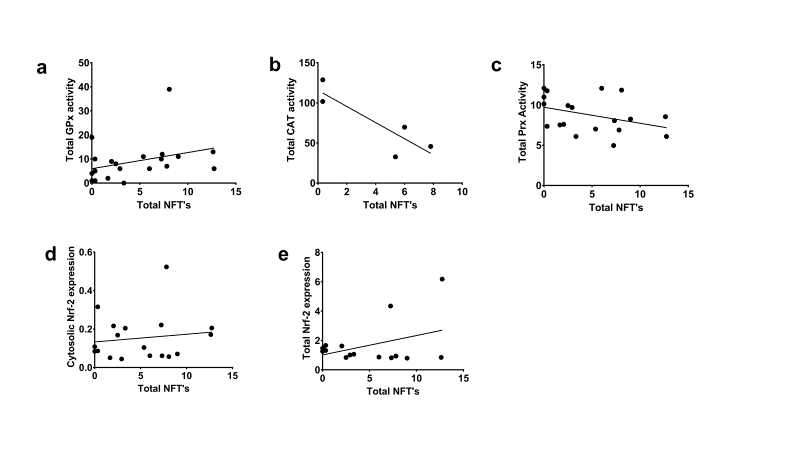
**Supplementary Figure S6**. Total NFTs correlated poorly (A) total GPx activity (B) total CAT activity (C) total Prx activity (D) cytosolic NRF-2 protein expression and (E) total Nrf-2 expression; data assessed using Prism *v*7.


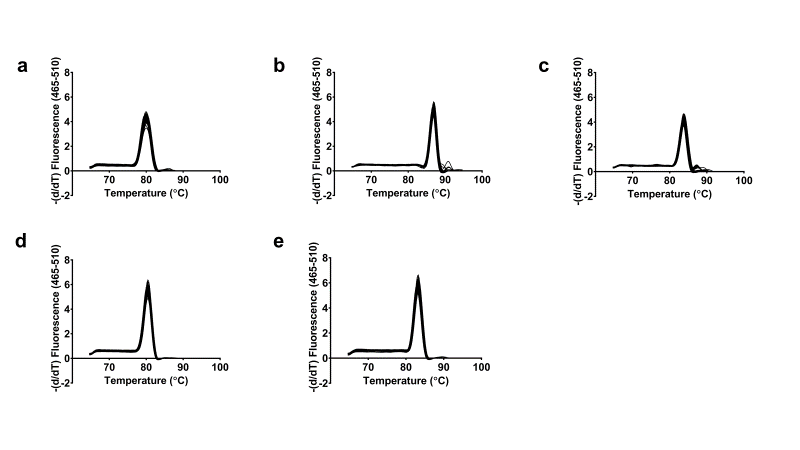


**Supplementary Figure S7**. Representative thermal melt curves confirming single PCR products were obtained for (A) the house-keeping gene SDHA, (B) HO-1, (C) HO-2 (D) NFE2L2, (E) SOD-1, when assessing gene regulation with Q-PCR as described in the Methods section in the corresponding main paper.

**Supplementary Table 1:** Summary of demographic and pathological comparisons. ^a^

| **Parameter (SD)** | **AD cases** | **Controls** | ***p*-Value** |
| --- | --- | --- | --- |
| Gender (M/F) | 10/11 | 10/9 | n/a |
| Mean age (years) | 77.6 (11.3) | 75.2 (10.6) | 0.50 |
| E3/E3, E3/E4 and E4/E4 | 10, 8 and 3 | 14, 5 and 0 | n/a |
| Mean PMI (h) | 14.0 (9.1) | 22.9 (15.6) | 0.0376* |
| Mean brain pH | 6.3 (0.2) | 6.6 (0.2) | <0.0001 |
| Mean RIN | 5.6 (1.2) | 6.7 (0.9) | 0.002** |
| Mean storage time (months) | 39.7 (25.0) | 53.2 (38.8) | 0.21 |
| Mean neuronal density (mm^2^) | 178.4 (38.8) | 205.8 (38.8) | 0.041* |
| Mean total NFTs (mm^2^) | 4.5 (4.2) | 0.0 (0.0) | <0.0001*** |
| AT8 tau-+ve neurons (mm^2^) | 35.0 (26.5) | 0.01 (0.03) | <0.0001*** |
| Plaques (areal fraction) | 0.03 (0.02) | 0.003 (0.01) | <0.0001*** |

^a^ Clinical data were collected for each subject donating tissue to the BioBank. Apolipoproteins E3/E4 = E3 and E4, respectively. PMI, post-mortem interval, RNA integrity number, RIN, * *p* <0.05,** *p* <0.01,*** *p* <0.0001
